# Neurally adjusted ventilatory assist and proportional assist ventilation both improve patient-ventilator interaction

**DOI:** 10.1186/s13054-015-0763-6

**Published:** 2015-02-25

**Authors:** Matthieu Schmidt, Felix Kindler, Jérôme Cecchini, Tymothée Poitou, Elise Morawiec, Romain Persichini, Thomas Similowski, Alexandre Demoule

**Affiliations:** Sorbonne Universités, UPMC Univ Paris 06, UMR_S 1158 Neurophysiologie Respiratoire Expérimentale et Clinique, F-75005 Paris, France; INSERM, UMR_S 1158 Neurophysiologie Respiratoire Expérimentale et Clinique, F-75005 Paris, France; AP-HP, Groupe Hospitalier Pitié-Salpêtrière Charles Foix, Service de Pneumologie et Réanimation Médicale (Département R3S), F-75013 Paris, France; Université Pierre et Marie Curie-CNRS-INSERM, ICM, Equipe Neurologie et Thérapeutique Expérimentale, Hôpital de la Salpêtrière, Paris, France; U974, Institut National de la Santé et de la Recherche médicale, Paris, France; Service de Pneumologie et Réanimation Médicale, Groupe Hospitalier Pitié-Salpêtrière, 47-83 boulevard de l’Hôpital, 75651 Paris, Cedex 13 France

## Abstract

**Introduction:**

The objective was to compare the impact of three assistance levels of different modes of mechanical ventilation; neurally adjusted ventilatory assist (NAVA), proportional assist ventilation (PAV), and pressure support ventilation (PSV) on major features of patient-ventilator interaction.

**Methods:**

PSV, NAVA, and PAV were set to obtain a tidal volume (V_T_) of 6 to 8 ml/kg (PSV_100_, NAVA_100_, and PAV_100_) in 16 intubated patients. Assistance was further decreased by 50% (PSV_50_, NAVA_50_, and PAV_50_) and then increased by 50% (PSV_150_, NAVA_150_, and PAV_150_) with all modes. The three modes were randomly applied. Airway flow and pressure, electrical activity of the diaphragm (EAdi), and blood gases were measured. V_T_, peak EAdi, coefficient of variation of V_T_ and EAdi, and the prevalence of the main patient-ventilator asynchronies were calculated.

**Results:**

PAV and NAVA prevented the increase of V_T_ with high levels of assistance (median 7.4 (interquartile range (IQR) 5.7 to 10.1) ml/kg and 7.4 (IQR, 5.9 to 10.5) ml/kg with PAV_150_ and NAVA_150_ versus 10.9 (IQR, 8.9 to 12.0) ml/kg with PSV_150_, *P* <0.05). EAdi was higher with PAV than with PSV at level_100_ and level_150_. The coefficient of variation of V_T_ was higher with NAVA and PAV (19 (IQR, 14 to 31)% and 21 (IQR 16 to 29)% with NAVA_100_ and PAV_100_ versus 13 (IQR 11 to 18)% with PSV_100_, *P* <0.05). The prevalence of ineffective triggering was lower with PAV and NAVA than with PSV (*P* <0.05), but the prevalence of double triggering was higher with NAVA than with PAV and PSV (*P* <0.05).

**Conclusions:**

PAV and NAVA both prevent overdistention, improve neuromechanical coupling, restore the variability of the breathing pattern, and decrease patient-ventilator asynchrony in fairly similar ways compared with PSV. Further studies are needed to evaluate the possible clinical benefits of NAVA and PAV on clinical outcomes.

**Trial registration:**

Clinicaltrials.gov NCT02056093. Registered 18 December 2013.

**Electronic supplementary material:**

The online version of this article (doi:10.1186/s13054-015-0763-6) contains supplementary material, which is available to authorized users.

## Introduction

Partial ventilatory assistance minimizes adverse effects of controlled mechanical ventilation, such as excessive sedation and ventilator-induced diaphragm dysfunction [1-3]. The most widely used partial ventilatory assistance mode is pressure support ventilation (PSV) [4], in which a constant preset level of pressure assists each inspiration, regardless of the patient’s inspiratory effort. Mismatching between patient demand and level of assistance is therefore possible and can be potentially harmful: underassistance may induce respiratory discomfort [5], and overassistance may cause lung overdistention and volutrauma [6]. Of note, underassistance and overassistance may both generate patient-ventilator asynchrony that is associated with poorer clinical outcomes [7].

Proportional Assisted Ventilation (PAV) and Neurally Adjusted Ventilatory Assist (NAVA) have been designed to overcome this weakness of PSV. These two modes adjust proportionally the amount of assistance delivered. NAVA adjusts ventilator assistance to the electrical activity of the diaphragm (EAdi), recorded with an esophageal catheter [8]. PAV adjusts ventilator assistance to the activity of respiratory muscles estimated by an algorithm [9]. Previous studies have shown the potential benefits of PAV and NAVA to prevent the risk of overassistance [10-13], to increase the variability of the breathing pattern [14-20], and to improve patient-ventilator interaction and synchrony [11,12,21-26]. PAV and NAVA have been previously compared with PSV but not with each other. This comparison would be clinically relevant, as these two modes have their own specific strengths and weaknesses [9,27].

In the study reported here, we hypothesized that PAV and NAVA improve patient-ventilator interaction in similar ways. The aim of this study was therefore to compare, in patients recovering from acute respiratory failure, the respective impacts of various levels of NAVA, PAV, and PSV on four major features of patient-ventilator interaction: (1) breathing pattern, including prevention of overassistance; (2) respiratory drive; (3) breathing pattern variability, and (4) patient-ventilator synchrony.

## Materials and methods

The study was conducted over a period of 3 months in a 10-bed Intensive Care Unit (ICU) in an 1,800-bed university hospital. The protocol was approved by the *Comite de Protection des Personnes* Ile de France VI. Informed consent was obtained from patients or relatives.

### Patients

Patients initially intubated and ventilated in the ICU were eligible for inclusion in the study if (1) they had been ventilated for acute respiratory failure *via* an endotracheal tube for more than 48 hours, (2) the condition that had required mechanical ventilation had improved (in particular, the ability to trigger the ventilator with an FiO_2_ of ≤50% and positive end-expiratory pressure (PEEP) ≤5 cmH_2_O), (3) sedation had been stopped for more than 6 hours, (4) hemodynamic stability was achieved without vasopressor or inotropic medication. Exclusion criteria were known or suspected phrenic nerve dysfunction or other neuromuscular disorders that may involve the diaphragm or impair respiratory drive. Patients with contraindications to EAdi catheter placement (for example, gastroesophageal varices or obstruction, recent gastroesophageal surgery, facial surgery or trauma, or upper gastrointestinal bleeding) were excluded. Patients in whom the decision had been made to withhold life-sustaining treatment were also ineligible for inclusion.

### Ventilation equipment

The conventional nasogastric tube was removed and replaced by a 16 Fr EAdi catheter (Maquet Critical Care, Solna, Sweden), and its position was controlled according to the manufacturer’s recommendations [28]. PSV and NAVA were delivered by using a Servo-I ventilator (Maquet Critical Care), and PAV+ was delivered by using a PB840 ventilator (Covidien, Boulder, CO, USA). Male and female patients were ventilated with an 8- and 7.5-mm internal diameter endotracheal tube, respectively.

### Study protocol

Inspiratory pressure support level was initially titrated to obtain a tidal volume (V_T_) of 6 to 8 ml/kg of predicted ideal body weight. Flow-trigger sensitivity was set at the lowest possible level without inducing autotriggering, and cycling-off was set at 30% of peak inspiratory flow (default value). This level of assistance was defined as PSV_100_. Patients were then switched to NAVA, and the corresponding NAVA level to obtain a similar V_T_ of 6 to 8 ml/kg was determined during a 5-minute period. This NAVA level was termed NAVA_100_. Patients were finally switched to PAV, and the percentage unloading (%Assist) was set also to obtain a similar V_T_ of 6 to 8 ml/kg. This %Assist corresponded to PAV_100_. In each of the three modes, the assist level was further decreased by 50%, corresponding to PSV_50_, NAVA_50_, and PAV_50_ and then increased by 50%, corresponding to PSV_150_, NAVA_150_, and PAV_150_. In the Results section, PSV_100,_ NAVA_100_, and PAV_100_ define a medium assistance level also termed level_100_; PSV_50,_ NAVA_50_, and PAV_50_ define a low assistance level, also termed level_50_; and PSV_150,_ NAVA_150_, and PAV_150_ define a high assistance level also termed level_150_. Of note, inspiratory pressure-support level in PSV_50_ could not be lower than 7 cmH_2_O. A high upper pressure limit at 45 cmH_2_O was set in PAV and NAVA.

Positive end-expiratory pressure (PEEP) and inspired oxygen fraction (FiO_2_) were maintained constant throughout the study period at the values in use before patient enrollment. The endotracheal tube was suctioned before the beginning of each trial. Each patient underwent three 30-minute trials, in each mode, consisting of 20-minute stabilization followed by 10-minute recording stored on a computer for further analysis. The three modes were applied in computer-generated random order. At the end of each trial, arterial blood was sampled for gas analysis (Radiometer ABL 330, Tacussel, Copenhagen, Denmark) via a catheter, and dyspnea was rated by using a visual analogue scale when possible.

### Data acquisition

Flow was measured with a heated Fleisch pneumotachograph, dead space 51 ml (Hans Rudolph, Kansas City, MO, USA) and airway pressure was measured by a pressure transducer (DP 15–32, Validyne, Northridge, CA, USA) for all modes. Digital EAdi signal was converted into an analog signal (National Instruments, Austin, TX, USA). During all three modes of ventilation, the EAdi waveform was simultaneously recorded with flow and airway pressure from the respective ventilator (see Additional file 1). All signals were digitized at a 100-Hz sampling rate (PowerLab/4SP, ADInstruments, Castle Hill, Australia) and recorded on a personal computer for subsequent analysis (Chart software, ADInstruments, Castle Hill, Australia).

### Data analysis

#### Respiratory Parameters and Breathing Pattern

Neural respiratory rate (RR), V_T_, duration of pneumatic inspiration (Ti), maximum EAdi, (EAdi_max_), area under the curve of EAdi during inspiratory time (EAdi_AUC_, integrated from baseline to peak), and the V_T_-(ml/kg)/Eadi_max_ ratio were calculated offline from the 10-minute airway flow and EAdi recordings. The coefficient of variation (standard deviation divided by the mean) for both flow (CV_VT_) and EAdi-related variables (CV_EAdimax_) was calculated. Maximum (P_max_) and mean inspiratory airway pressure (P_mean_) were measured and calculated from airway pressure recordings.

#### Patient-ventilator interaction

Within the three modes and in all conditions, correlations between EAdi_max_ and P_max_ and between EAdi_AUC_ and V_T_ were calculated. The inspiratory trigger delay was measured as the time difference between the beginning of the increase in the EAdi signal and the beginning of the ventilator inspiratory flow. The expiratory trigger delay was measured as the time difference between EAdi_max_ and the end of the insufflation, as defined by a ventilator inspiratory flow equal to zero. Using the EAdi waveform, we quantified the three main types of asynchronies accordingly to previously published definitions [7,25] (see also Additional file 2): (1) ineffective efforts; (2) auto-triggering, and (3) double triggering. Of note, only type II double triggering, defined as one neural inspiration triggering two breath cycles, was considered [25] (see example in Additional file 3). The number of each type of asynchrony was reported as the total number of each event per minute. A global asynchrony index (AI) was computed [7].

### Statistical analysis

Statistical analysis was performed with Prism 4.01 software (GraphPad Software, San Diego, CA, USA). Normality testing failed for all results (Kolmogorov-Smirnov). Results are therefore expressed as median (25 to 75 interquartile range). Within each of the three assistance level groups (that is, level_50_, level_100_, and level_150_), Friedman ANOVA for repeated measures was performed to compare breathing pattern, variability, prevalence of the main asynchronies and blood gases measured with PAV, NAVA, and PSV, respectively. Comparison between the three modes was followed, when appropriate, by a pairwise comparison by using the Dunn *post/hoc* test. The relationships among both EAdi_max_ and P_max_ and EAdi_AUC_ and V_T_ were examined by using a linear regression analysis, and the coefficient of correlation (r^2^) was determined. Differences were considered significant when the probability p of a type I error was less than 5%.

## Results

The study pertains to a convenience sample of 16 patients (10 males). Their main characteristics and the precipitating factor of acute respiratory failure are summarized in Table 1. Respective assistance levels used for each mode are reported in Table 2. Of note, three patients had chronic obstructive pulmonary disease (COPD) (patients 5, 7, and 16).

**Table 1:** **Patient characteristics at enrollment**

**Patient no.**	**Age (years)**	**BMI (kg.m** ^**−2**^ **)**	**SAPS 2**	**Admission diagnosis**	**MV duration before inclusion (days)**	**FiO**_**2**_	**PEEP (cmH**_**2**_ **O)**
1	70	32.0	61	Pneumonia	8	0.5	4
2	90	29.9	63	ARDS	2	0.4	5
3	83	16.0	55	ARDS	6	0.5	4
4	67	37.6	56	Pneumonia	12	0.5	5
5	77	27.8	47	Acute respiratory failure due to decompensation of COPD	7	0.5	4
6	60	27.5	35	Pneumonia	8	0.5	4
7	60	25.0	26	Acute respiratory failure due to decompensation of COPD	7	0.5	4
8	81	33.2	49	Pneumonia	4	0.4	4
9	74	18.4	63	Pneumonia	6	0.5	4
10	64	23.5	97	Pneumonia	11	0.5	5
11	67	23.0	51	ARDS	12	0.5	4
12	62	22.8	84	ARDS	7	0.5	4
13	62	25.0	49	Pneumonia	2	0.5	5
14	66	29.4	67	Acute respiratory failure due to decompensation of COPD	21	0.5	5
15	65	26.0	72	Pneumonia	12	0.5	5
16	67	31.6	46	Pneumonia	2	0.4	5
**Median (IQR)**	**67 (63–75)**	**27 (23–30)**	**55 (48–64)**		**7.0 (5.5-11.0)**	**0.5 (0.5-0.5)**	**4 (4–5)**

M, male; F, female; BMI, body mass index; SAPS II, Simplified Acute Physiology Score II; MV, mechanical ventilation; PEEP, positive end-expiratory pressure; IQR, interquartile range; ARDS, acute respiratory distress syndrome, COPD, chronic obstructive pulmonary disease.

**Table 2:** **Assistance levels in each experimental condition**

**Mode, assistance setting**	**Level**_**50**_	**Level**_**100**_	**Level**_**150**_	**Notes**
PSV, inspiratory pressure (cmH_2_O)	7.0 (7.0-7.2)	14.0 (11.5-15.2)	21.0 (17.2-21.7)	The inspiratory pressure support level, set by the clinician, is kept constant regardless of the mechanical properties of the lung/thorax and patient effort.
NAVA, NAVA level (cmH_2_O/μV)	0.6 (0.4-0.9)	1.3 (0.8-1.8)	1.9 (1.2-2.7)	The NAVA level is a proportional gain factor expressed in cmH_2_O/μV of EAdi. It represents the magnitude (in cmH_2_O) of positive airway pressure applied per μV EAdi during the course of each inspiration.
PAV, proportion of assistance (%)	27 (25–35)	55 (50–70)	82 (75–95)	The proportion of assistance is the percentage of work provided by the ventilator. The rest of the work is provided by the patient.

EAdi, electrical activity of the diaphragm; PSV, pressure support ventilation; NAVA, neurally adjusted ventilatory assist; PAV, proportional assist ventilation.

Data are provided as median (interquartile range).

### Breathing pattern and electrical activity of the diaphragm

**Figure 1:**
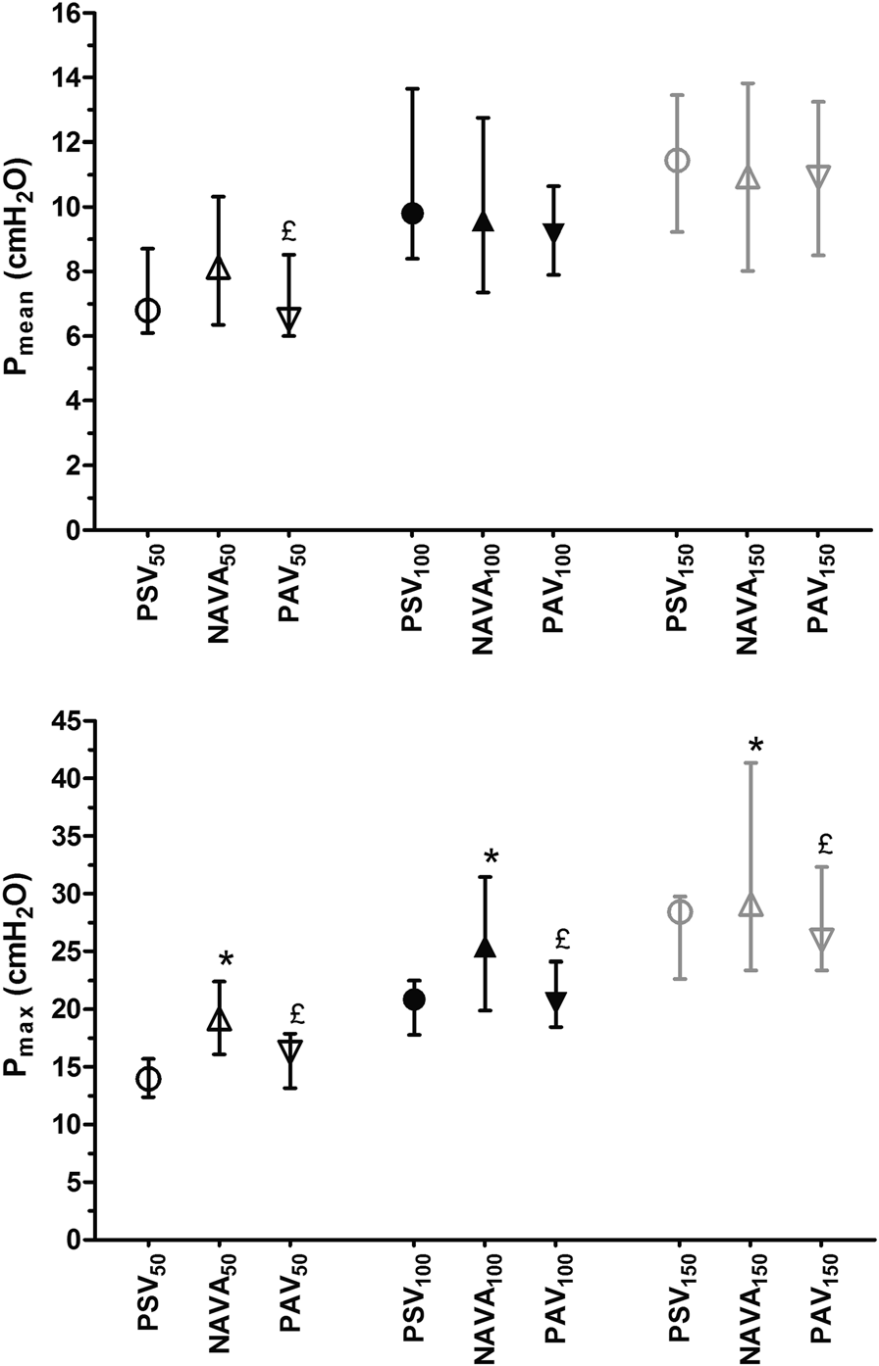
**Impact of ventilator mode and level of assistance on mean (** ***P***_***mean***_ **) and maximum airway pressure (** ***P***_***max***_ **).** Pressure support ventilation (PSV)_100_, neurally adjusted ventilatory assist (NAVA)_100_, and proportional assist ventilation (PAV)_100_ are medium levels of assistance set to obtain a tidal volume (V_T_) between 6 and 8 ml/kg ideal body weight. PSV_50_, NAVA_50_ and PAV_50_ are low levels of assistance defined by decreasing the assistance level by 50% in each condition. Inversely, PSV_150_, NAVA_150_, and PAV_150_ are defined by increasing the assistance level by 50% in each condition. *P <0.05 with PSV; ^£^ *P* <0.05 with NAVA. Data are expressed as median and interquartile range.

**Figure 2:**
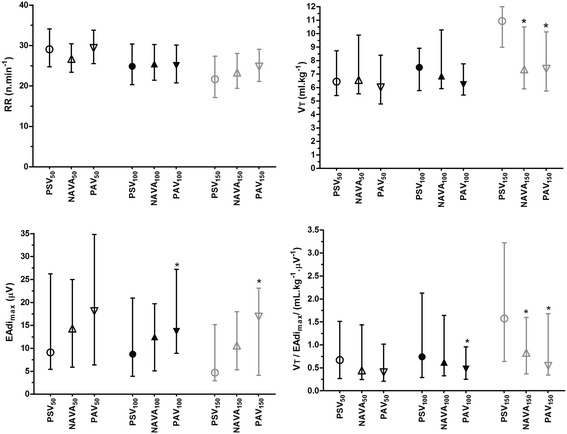
**Impact of ventilator mode and level of assistance on the major descriptors of breathing pattern and diaphragmatic electrical activity (EAdi).** Pressure support ventilation (PSV)_100_, neurally adjusted ventilatory assist (NAVA)_100_, and proportional assist ventilation (PAV)_100_ are medium levels of assistance set to obtain a tidal volume (V_T_) between 6 and 8 ml/kg ideal body weight. PSV_50_, NAVA_50_ and PAV_50_ are low levels of assistance defined by decreasing the assistance level by 50% in each condition. Inversely, PSV_150_, NAVA_150_, and PAV_150_ are defined by increasing the assistance level by 50% in each condition. *EAdi*_*max*_ *,* peak of EAdi; *RR*, respiratory rate; *V*_*T*_ */Eadi*_*max*_, neuromechanical coupling. *P <0.05 with PSV. Data are expressed as median and interquartile range.

Group median values for representative breathing pattern variables are provided in Figure 1 and Figure 2 (see also Additional file 4). Inspiratory pressures for each patient under all conditions are displayed in Additional file 4. Median airway pressure was similar among level_100_ and level_150_ groups. Within all assistance levels (level_50_, level_100_, level_150_), P_max_ was higher with NAVA than with PAV and PSV (*P* = 0.001; Figure 1, Table 3). At level_50_ and level_100_, V_T_ was similar among modes. However, at a high assistance level, V_T_ was significantly higher with PSV_150_ than with NAVA_150_ and PAV_150_ (*P* < 0.05). Tidal volume was similar with NAVA and PAV regardless of the assistance level. Inspiratory time and RR remained similar within all modes and at each level of assistance. Of note, at level_100,_ and level_150_, EAdi_max_ and EAdi_AUC_ were higher in PAV than in PSV (Figure 2; Table 3). Whereas the V_T_/EAdi_max_ ratio was similar among groups at level_50_, it was higher with PSV than with PAV at level_100_ and level_150_ (*P* <0.0001). In addition, the V_T_/EAdi_max_ ratio was higher with PSV (*P* <0.0001) but did not differ between PAV and NAVA regardless of the assistance level.

**Table 3:** **Impact of ventilator mode and level of assistance on the main descriptors of breathing pattern and electrical activity of the diaphragm (EAdi)**

	**PSV**	**NAVA**	**PAV**
P_mean_ (cmH_2_O)			
level_50_	6.8 (6.1-8.7)	8.1 (6.3-10.3)	6.5 (6.0-8.5)^£^
level_100_	9.8 (8.4-13.6)	9.6 (7.3-12.7)	9.1 (7.9-10.6)
level_150_	11.4 (9.2-13.4)	10.9 (8.0-13.8)	10.9 (8.5-13.2)
P_peak_ (cmH_2_O)			
level_50_	13.9 (12.4-15.7)	19.2 (16.1-22.4)*	16.2 (13.1-17.9)^£^
level_100_	20.8 (17.8-22.5)	25.4 (19.9-31.4)*	20.5 (18.5-24.1)^£^
level_150_	28.4 (22.6-29.7)	29.1 (23.4-41.3)*	25.9 (23.4-32.3)^£^
Respiratory rate (n.min^−1^)			
level_50_	30 (25–34)	28 (25–32)	30 (26–34)
level_100_	24 (19–32)	27 (22–30)	28 (17–31)
level_150_	23 (17–25)	25 (20–29)	26 (18–30)
Tidal volume (mL.kg^−1^ IBW)			
level_50_	6.4 (5.4-8.7)	6.6 (5.5-9.9)	6.0 (4.8-8.4)
level_100_	7.5 (5.8-8.9)	6.9 (5.9-10.3)	6.2 (5.4-7.8)
level_150_	10.9 (8.9-12.0)	7.4 (5.9-10.5)*	7.4 (5.7-10.1)*
Minute ventilation (mL.min^−1^)			
level_50_	10.8 (8.9-15.8)	9.8 (8.2-14.4)	11.0 (7.9-14.5)
level_100_	10.6 (8.2-14.1)	10.0 (9.2-16.6)	9.8 (8.0-13.5)
level_150_	12.5 (9.8-17.3)	10.1 (7.8-13.9)	11.3 (8.6-14.5)
Inspiratory time (sec)			
level_50_	0.72 (0.67-0.82)	0.77 (0.60-0.84)	0.73 (0.61-0.99)
level_100_	0.72 (0.67-0.95)	0.78 (0.61-0.93)	0.76 (0.64-1.06)
level_150_	0.85 (0.69-1.18)	0.79 (0.59-0.93)	0.83 (0.64-1.09)
EAdi_max_			
level_50_	9.1 (5.4-26.2)	14.3 (5.9-25.0)	18.1 (6.4-34.8)
level_100_	8.7 (3.9-20.9)	12.5 (5.1-19.7)	13.7 (8.9-27.2)*
level_150_	4.7 (2.9-15.1)	10.6 (5.3-18.0)	10.9 (4.1-13.1)*
EAdi_AUC_			
level_50_	9.5 (3.9-13.6)	9.9 (4.8-16.0)	13.2 (4.7-19.9)
level_100_	7.1 (3.6-13.5)	8.5 (4.9-12.3)	12.2 (6.2-18.8)*
level_150_	4.3 (2.4-8.7)	8.9 (4.4-11.4)	11.4 (3.3-14.5)*
V_T_ /EAdi_max_			
level_50_	0.67 (0.27-1.51)	0.44 (0.24-1.43)	0.40 (0.20-1.01)
level_100_	0.74 (0.29-2.13)	0.62 (0.33-1.64)	0.47 (0.25-0.96)*
level_150_	1.67 (0.64-3.22)	0.83 (0.37-1.60)*	0.54 (0.34-1.68)*

EAdi_max_, peak EAdi; EAdi_AUC_, area under the EAdi curve; P_mean_; mean inspiratory airway pressure; P_max_, maximum inspiratory airway pressure; PSV, pressure support ventilation; PAV, proportional assist ventilation; NAVA, neurally adjusted ventilatory assist.

Level_100_ is a medium assistance level set to obtain a VT of 6 to 8 ml/.kg ideal body weight. Level_50_ is a low assistance level defined as level_100_ decreased by 50%. Level_150_ is a high assistance level defined as level_100_ increased by 50%.

Data are provided as median (interquartile range).

*P <0.05 with PSV; ^£^ *P* <0.05 with NAVA.

### Breath-by-breath variability

**Figure 3:**
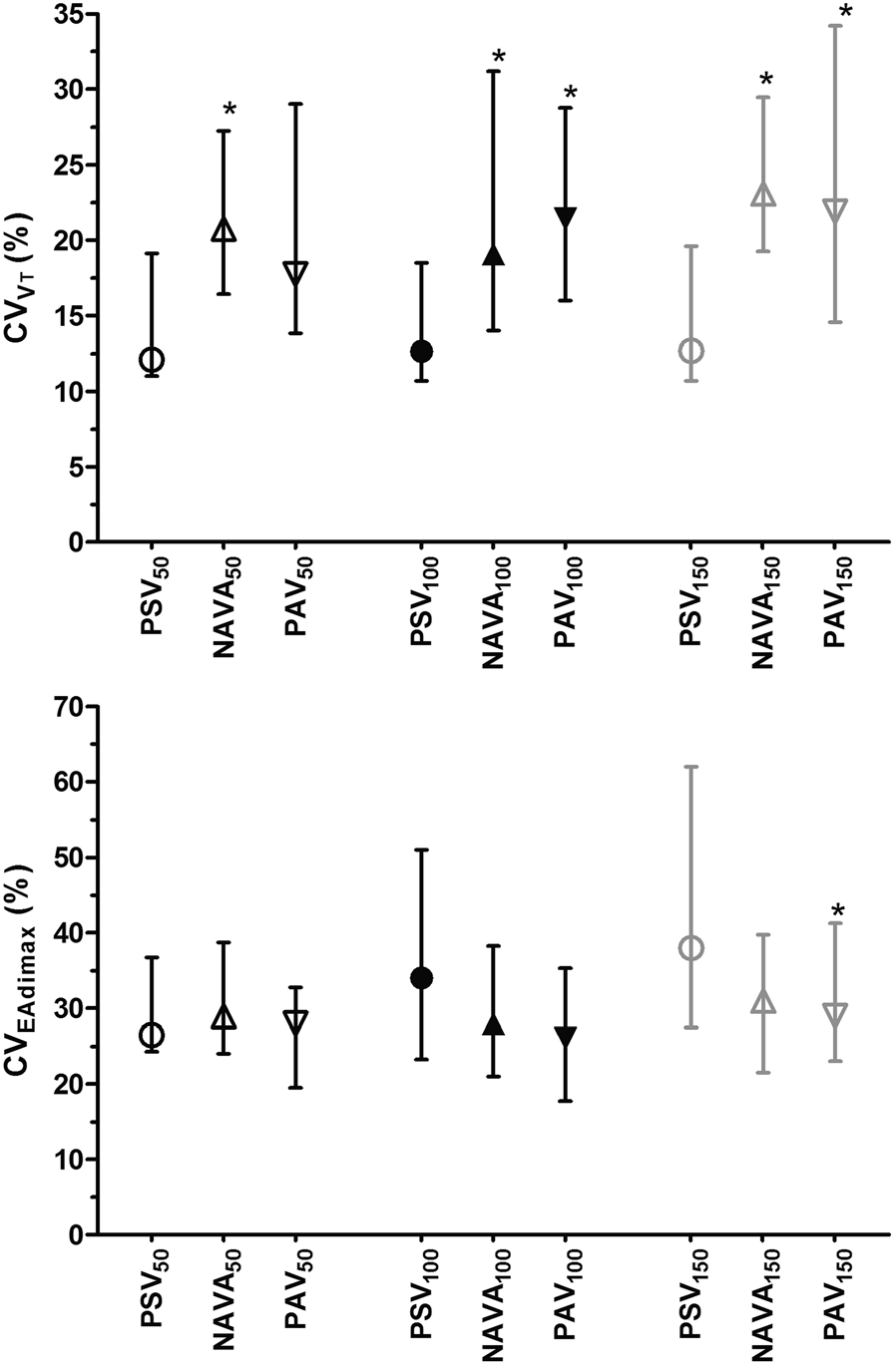
**Impact of ventilator mode and level of assistance on the coefficient of variation of tidal** ***volume*** **(CV**_**VT**_ **) and maximum electrical activity of the diaphragm (CV**_**EAdimax**_ **).** Pressure support ventilation (PSV)_100_, neurally adjusted ventilatory assist (NAVA)_100_, and proportional assist ventilation (PAV)_100_ are medium levels of assistance set to obtain a tidal volume (V_T_) between 6 and 8 ml/kg ideal body weight. PSV_50_, NAVA_50_ and PAV_50_ are low levels of assistance defined by decreasing the assistance level by 50% in each condition. Inversely, PSV_150_, NAVA_150_ and PAV_150_ are defined by increasing the assistance level by 50% in each condition. *P <0.05 with PSV; ^£^ *P* <0.05 with NAVA; Data are expressed as median and interquartile range.

Group median values for coefficient of variation V_T_ and EAdi_max_ are provided in Figure 3 (see also Additional file 5). The coefficient of variation of V_T_ was higher with PAV and NAVA than with PSV at level_100_ and level_150_ (*P* <0.05), whereas the coefficient of variation of V_T_ was similar between NAVA and PAV at each level of assistance. Conversely, the coefficient of variation of EAdi_max_ did not change according to ventilator mode and level of assistance, except at level_150_, where it was lower with PAV_150_ than with PSV_150_.

### Patient-ventilator interaction

Table 4 and Additional file 6 show the inspiratory and expiratory trigger delays, the correlation between both V_T_ and P_max_ and EAdi, and the prevalence of patient-ventilator asynchrony in each condition. Inspiratory trigger delay was significantly lower in NAVA than in PAV and PSV at level_100_ and level_150_, respectively. Similarly, expiratory trigger delay was lower during NAVA_100_ and NAVA_150_ than during PSV_100_ and PSV_150_, respectively (Table 4). The correlation between EAdi_AUC_ and V_T_, was higher during NAVA and PAV than during PSV (Table 4). The correlation between EAdi_max_ and P_max_ was higher during NAVA than during PAV and PSV (Table 4). At each level of assistance, almost no ineffective efforts were reported with PAV and NAVA, whereas the ineffective efforts were detected with PSV at a higher level (*P* <0.05). Inversely, although very few double-triggering events were observed with PSV and PAV, the prevalence of double triggering was significantly higher with NAVA (*P* <0.05, Table 4). Type II double triggering was due to ventilator cycled off when the EAdi dropped to 70% of its peak, followed by a rebound in inspiratory flow, cause of the retriggering, when cycled off to PEEP (see Additional file 3).

**Table 4:** **Impact of ventilator mode and level of assistance on patient-ventilator interaction and asynchrony indices**

	**PSV**	**NAVA**	**PAV**
Inspiratory trigger delay (msec)			
level_50_	162 (109–241)	157 (138–289)	185 (140–305)
level_100_	170 (140–282)	124 (100–238)	224 (176–280)^£^
level_150_	266 (140–427)	164 (99–278)*	201 (166–317)
Expiratory trigger delay (msec)			
level_50_	156 (112–191)	151 (125–175)	152 (107–175)
level_100_	212 (176–273)	157 (140–188)*	182 (116–230)
level_150_	255 (227–541)	146 (131–218)*	225 (169–333)
Relationship between P_max_ and EAdi_max_ (r^2^)			
level_50_	0.02 (0.01-0.04)	0.56 (0.51-0.65)*	0.02 (0.00-0.04)^£^
level_100_	0.03 (0.01-0.08)	0.85 (0.78-0.90)*	0.04 (0.00-0.07)^£^
level_150_	0.03 (0.01-0.06)	0.64 (0.43-0.88)*	0.03 (0.02-0.08)^£^
Relationship between V_T_ and EAdi_AUC_ (r^2^)			
level_50_	0.15 (0.14-0.19)	0.48 (0.40-0.57)*	0.39 (0.25-0.45)*
level_100_	0.13 (0.06-0.22)	0.64 (0.60-0.79)*	0.29 (0.21-0.42)
level_150_	0.02 (0.01-0.06)	0.72 (0.65-0.88)*	0.28 (0.25-0.41)*
Ineffective efforts (n.min^−1^)			
level_50_	0.00 (0.00-0.07)	0.00 (0.00-0.00)*	0.00 (0.00-0.00)*
level_100_	0.03 (0.00-0.26)	0.00 (0.00-0.00)*	0.00 (0.00-0.00)*
level_150_	0.27 (0.01-1.23)	0.00 (0.00-0.00)*	0.00 (0.00-0.00)*
Auto-triggering (n.min^−1^)			
level_50_	0.00 (0.00-0.03)	0.00 (0.00-0.00)	0.00 (0.00-0.00)
level_100_	0.00 (0.00-0.03)	0.00 (0.00-0.00)	0.00 (0.00-0.00)
level_150_	0.00 (0.00-0.00)	0.00 (0.00-0.00)	0.00 (0.00-0.00)
Double triggering (n.min^−1^)			
level_50_	0.00 (0.00-0.00)	0.42 (0.08-0.50)*	0.00 (0.00-0.00)^£^
level_100_	0.00 (0.00-0.18)	0.33 (0.10-0.92)*	0.00 (0.00-0.10)^£^
level_150_	0.00 (0.00-0.21)	0.30 (0.02-0.87)*	0.00 (0.00-0.20)^£^
Asynchrony index (%)			
level_50_	0.21 (0.00-0.65)	1.41 (0.34-2.91)*	0.13 (0.00-0.44)^£^
level_100_	0.61 (0.04-1.28)	1.73 (0.38-2.69)	0.00 (0.00-0.58)^£^
level_150_	1.65 (0.58-5.77)	1.17 (0.05-3.99)	0.19 (0.00-1.12)

EAdi_AUC_, area under the diaphragmatic electrical activity curve; PSV, pressure support ventilation; NAVA, neurally adjusted ventilatory assist; PAV, proportional assist ventilation; VT, tidal volume. Level_100_ is a medium assistance level set to obtain a VT of 6to 8 ml/kg ideal body weight. Level_50_ is a low assistance level defined as level_100_ decreased by 50%. Level_150_ is a high assistance level defined as level_100_ increased by 50%.

The number of each type of asynchrony is reported as the total number of each event per minute. Asynchrony index is defined as the total number of asynchrony events × 100/(ventilator respiratory rate + ineffective efforts).

*P <0.05 with PSV; ^£^ *P* <0.05 with NAVA; data are expressed as median (interquartile range).

No autotriggering was observed in any condition. Overall, the asynchrony index was significantly lower with PAV_50_ and PAV_100_ than with NAVA_50_ and NAVA_100_, respectively (P < 0.05). Of note, only two patients exhibited an AI >10% in PSV_150_, mostly due to a high number of ineffective efforts (patients 7 and 14). Dyspnea was able to be evaluated in only two patients because of insufficient cooperation (data not shown).

### Gas exchange

Neither the mode (PSV, NAVA, PAV) nor the level of assistance (level_50, 100, 150_) influenced PaO_2_, PaCO_2_, or pH, which remained not significantly different between all conditions, except for PaCO_2_ that was higher and pH that was lower with PAV_100_, than with NAVA_100_ (see Additional file 7 for detailed blood gas values).

## Discussion

The main findings of our study are as follows: (1) PAV and NAVA both prevented overassistance-induced hyperinflation, in contrast with PSV; (2) PAV and NAVA restored a comparable level of breathing-pattern variability that was greater than the variability observed with PSV; (3) Regardless of the level of assistance, PAV and NAVA induced less patient-ventilator asynchrony than PSV, with the exception of double triggering, which was more frequent with NAVA. The similarities observed between NAVA and PSV in terms of breathing pattern, variability, and asynchrony are consistent with the conceptual similarities of these two modes.

### Breathing pattern and central respiratory neural output

Increasing PSV assist levels were associated with increasing V_T_ values, in keeping with previous data [29,30]. In contrast, V_T_ remained stable with NAVA and PAV, despite increasing assist levels [12,31], suggesting that these modes protect against overdistention. With PSV, the end of the patient’s inspiratory effort does not determine cycling-off of the ventilator. A patient may therefore trigger a PSV breath with a small inspiratory effort, then relax, and be passively insufflated. If this breath is given at an excessive assist level, the insufflation may continue while the patient has already stopped inspiring. In contrast, with PSV, NAVA and PAV deliver an insufflation that stops when either the output of the inspiratory centers to the diaphragm ends, in the case of NAVA (12), or when the inspiratory muscle activity ends, in the case of PAV.

In addition, because overdistention contributes to downregulate the activity of respiratory control centers (29), tidal volume is maintained constant with PAV and NAVA but not with PSV. The robustness of this protective biofeedback provided by proportional modes, as opposed to PSV, is illustrated in the present study by the marked alteration of the coupling between V_T_ and EAdi_*max*_ (that is, higher V_T_ /EAdi ratio) observed with PSV at high levels of assistance (see Figure 2), which was not observed with the two proportional modes.

### Breath-by-breath variability

Fluctuations in the resting breathing pattern of healthy humans have been known for a long time [32]. Breathing pattern variability seems to originate from the activity of central pattern generators [33]. It is further influenced by the load–capacity relationship of the respiratory system: the higher the loading, the lower the variability [19,34,35].

In the present study, the variability of V_T_ with NAVA and PAV was greater than with PSV at each assistance level. In contrast, the variability of EAdi was similar between the three modes, except at high assistance level. These data indicate that the increase in breath-to-breath variability observed during NAVA and PAV is actually due to “unmasking” of the underlying variability in central respiratory neural output and is a direct result of improvement of neuromechanical coupling. To our knowledge, these data, previously described in NAVA [19], have never been described with PAV. They suggest that PAV and NAVA both improve neuromechanical coupling in similar ways.

### Patient-ventilator interaction

As previously observed, NAVA and PAV improved patient-ventilator synchrony as compared with PSV [12,21,22,24,25,31]. Although inspiratory trigger delays in all modes were consistently greater than previously reported [11,36,37], lower inspiratory and expiratory trigger delays seemed to be more frequently noted in NAVA. Wide variability of the delays (see Additional file 6) and their greater values can be ascribed to different ventilators used, varying levels of assist provided, experimental settings themselves, and the different etiologies of respiratory failure. It is noteworthy that, in the present study, PAV and NAVA provided a similar benefit on ineffective triggering. It suggests that PAV and NAVA improve the relationship between EAdi and tidal volume in a similar way, which in turn prevents chest hyperinflation, a major risk factor for ineffective triggering [7].

Two types of double triggering have been described in NAVA [25]. Type I double triggering is the result of a biphasic EAdi signal, but its significance is unknown, which is why, strictly speaking, it cannot be considered to be patient–ventilator asynchrony. With type II double triggering, however, one neural inspiration triggers two breaths, which was due to ventilator cycle off when the EAdi dropped to 70% of its peak, followed by a rebound in inspiratory flow, cause of the re/-triggering, when cycled off to PEEP. Pneumatic trigger set to pressure instead of flow might limit the rebound in inspiratory flow. We therefore considered only type II double triggering in the present study and observed that this asynchrony was significantly more frequent with NAVA than with PSV and PAV. The relevance of this asynchrony and how to decrease its prevalence in NAVA need further investigations [25].

The correlation between V_T_ and EAdi_AUC_ was much weaker in PSV than in NAVA or PAV, whereas no significant difference was found between NAVA and PAV, which demonstrates that these two modes provide an assistance that is proportional to the central respiratory drive. This is consistent with the recent report from Akoumianaki *et al.* [38], showing that the correlation between the inspiratory integral of transdiaphragmatic pressure and the V_T_ was weaker with NAVA than with PAV [38]. Interestingly, the correlation between EAdi_max_ and P_max_ was higher in NAVA than in PAV, which may have two distinct explanations. First, during NAVA, EAdi and airway pressure are by definition strictly proportional and a strong correlation between EAdi_max_ and P_max_ is intrinsic to NAVA. Second, as opposed to NAVA that delivers an assistance proportional to the only diaphragm activity, PAV delivers an assistance that is proportional to the whole inspiratory activity of respiratory muscles. As a consequence, PAV integrates not only diaphragm activity, but also the activity of extradiaphragmatic inspiratory muscles such as scalenes or parasternal intercostal muscles [39].

### Limitations of the study

Our study has several limitations. First, as patients at high risk of asynchrony (for example, difficult-to-wean or severe COPD patients) were not specifically selected in this study [7] and because we targeted a V_T_ of 6 to 8 ml/kg in level_100_ [40], a very low incidence of asynchrony was observed with all modes and conditions. This study may therefore have underestimated the benefits of NAVA and PAV [20,39,41,42], but we deliberately decided to compare these modes in patients in the recovery phase after acute respiratory failure encountered in daily practice rather than in a very selected population, with the risk of showing results that would be transposable only to a niche population.

Second, the trials in our study were probably not sufficiently long to allow an improvement of gas exchange. This might explain why, despite a greater variability of the breathing pattern in PAV and NAVA, no impact on PaO_2_ was observed in contrast with previously published results [14].

Third, the choice of a resulting V_T_ of 6 to 8 ml/kg to match the assistance level_100_ with the three modes may be questionable. Indeed, a poor correlation between V_T_ and PAV %Assist [43] as well as NAVA level [18] has been reported. In addition, the high V_T_ variability may have jeopardized the accuracy of its setting. However, the fact that we observed a comparable P_mean_ with the three modes at assistance level_100_ suggests that the patients received a comparable level of assistance.

Fourth, because we focused on patients in the recovery phase after acute respiratory failure and because PSV_50_ could not be lower than 7 cmH_2_O, PSV_100_ settings could sometimes be very close to PSV_50_.

Fifth, although the expiration starts at 70% of the EAdi_max_ in NAVA, the expiratory trigger delay was calculated as the time difference between EAdi_max_ and the end of insufflation by the ventilator within the three modes. Finally, contrary to the sequence of the ventilatory modes tested, the sequence of the level of assistance was not randomized. Therefore, we cannot rule out a potential time effect.

### Clinical implications

Most of our findings are potentially clinically relevant. Lung-protective ventilation has become a major concern in ICU patients, even in those without acute respiratory distress syndrome [44,45]. Preventing alveolar overdistention and subsequent volotrauma caused by lung hyperinflation is now a major therapeutic goal. In this respect, NAVA and PAV provide an interesting tool to prevent overassistance-induced hyperinflation.

Variability of breathing pattern has become a matter of concern in ICU patients, as a recent study showed that a higher variability of respiratory rate was associated with better prognosis [46]. In addition, a more variable breathing pattern is associated with better pulmonary function in animal models of lung injury [47-51]. Finally, severe patient-ventilator asynchrony is associated with longer duration of mechanical ventilation and a greater need for tracheostomy [7]. Of note, patient-ventilator asynchrony may be either a cause or a consequence of the severity of the respiratory disease requiring mechanical ventilation. Whether optimization of ventilatory settings, by using PAV or NAVA, can shorten the duration of mechanical ventilation by reducing the incidence of asynchrony, has therefore not been demonstrated.

## Conclusion

In conclusion, PAV and NAVA both prevent overdistention and improve neuromechanical coupling and patient-ventilator asynchrony in fairly similar ways compared with PSV. Further studies are needed to evaluate the possible clinical benefits of NAVA and PAV on clinical outcomes, especially in the recovery phase of acute respiratory failure.

## Key messages

- The variability of V_T_ with NAVA and PAV is greater than with PSV at each assistance level.
- PAV and NAVA both restore “natural” variability of breathing.
- The increase in breath-to-breath variability observed during NAVA and PAV is due to “unmasking” of the underlying variability in central respiratory neural output and is a direct result of improvement of neuromechanical coupling.
- NAVA and PAV both improve patient-ventilator synchrony as compared with PSV, especially on ineffective triggering.

## Additional files

Additional file 1:
**Waveforms of EAdi, pressure and flow for all three modes (PSV**_**100**_ **, NAVA**_**100**_ **, PAV**_**100**_ **) in the same patient.**
Additional file 2:
**Definitions of patient- ventilator interaction indices and the main asynchronies collected.**
Additional file 3:
**Example of Type II double triggering under NAVA.**
Additional file 4:
**Inspiratory pressure (cmH**_**2**_ **O) over PEEP for each patient under the three modes and three assist levels.**
Additional file 5:
**Impact of ventilator mode and assistance level on the coefficients of variation of neural respiratory rate, tidal volume, and peak electrical activity of the diaphragm (EAdi**_**max**_ **).**
Additional file 6:
**Distribution of the inspiratory trigger delays per mode.**
Additional file 7:
**Impact of ventilator mode and level of assistance on gas exchange.**

## Abbreviations

AI: Asynchrony index
CV: coefficient of variation
CV_EAdimax_: coefficient of variation of EAdi_max_
EAdi: electrical activity of the diaphragm
EAdi_max_: maximum electrical activity of the diaphragm
EAdi_AUC_: integrated EAdi activity
IQR: interquartile range
NAVA: neurally adjusted ventilatory assist
PAV: proportional assisted ventilation
PEEP: positive end-expiratory pressure
P_max_: peak airway pressure
PSV: pressure support ventilation
RR: respiratory rate
Ti: inspiratory time
VT: tidal volume
