# Supplementary material for: Neurally adjusted ventilatory assist and proportional assist ventilation both improve patient-ventilator interaction
Source: Crit Care. 2015 Feb 25;19(1):56. doi: 10.1186/s13054-015-0763-6 (PMC4355459; doi:10.1186/s13054-015-0763-6)
Supplement: Additional file 1: — Waveforms of EAdi, pressure and flow for all three modes (PSV 100 , NAVA 100 , PAV 100 ) in the same patient. [file 13054_2015_763_MOESM1_ESM.doc]

**Additional File 1. W**aveforms of EAdi, pressure and flow for all three modes (PSV_100_, NAVA_100_, PAV_100_) in a same patient.


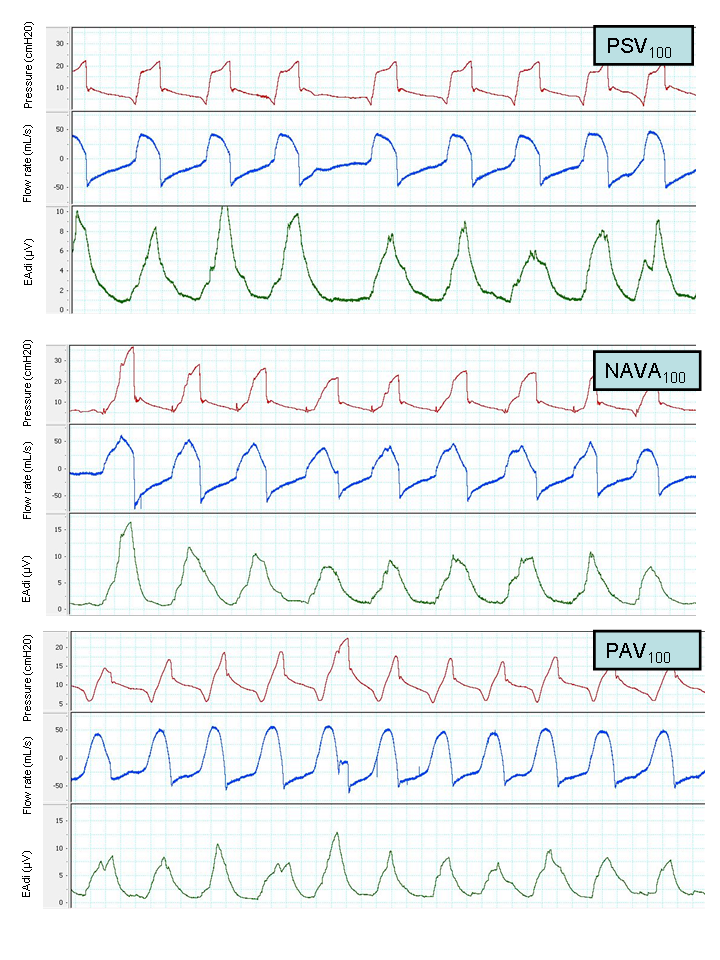


*EAdi,* electrical activity of the diaphragm; *PSV,* pressure support ventilation*; NAVA,* neurally adjusted ventilatory assist; *PAV*, proportional assist ventilation.
