# Supplementary material for: Neurally adjusted ventilatory assist and proportional assist ventilation both improve patient-ventilator interaction
Source: Crit Care. 2015 Feb 25;19(1):56. doi: 10.1186/s13054-015-0763-6 (PMC4355459; doi:10.1186/s13054-015-0763-6)
Supplement: Additional file 2: — Definitions of patient- ventilator interaction indices and the main asynchronies collected. [file 13054_2015_763_MOESM2_ESM.doc]

**Additional File 2. Definitions of patient- ventilator interaction indices and the main asynchronies collected**

| **Type of asynchrony** | **Definitions** |
| --- | --- |
| Inspiratory trigger delay | Time difference between the beginning of the increase in the EAdi signal and the beginning of the ventilator inspiratory flow |
| Ineffective effort | Presence of a characteristic electrical activity of the diaphragm not followed by a ventilator-delivered breath |
| Auto-triggering | A ventilator-delivered breath with no corresponding electrical activity of the diaphragm |
| Double triggering | The occurrence of two ventilator-delivered breaths separated by an expiratory time less than half of the mean inspiratory time during one single electromyographic activity. |
| Asynchrony index | (Ineffective efforts + auto-triggering + double triggering) x 100 / (number of breaths + ineffective efforts). |
