# Supplementary material for: Neurally adjusted ventilatory assist and proportional assist ventilation both improve patient-ventilator interaction
Source: Crit Care. 2015 Feb 25;19(1):56. doi: 10.1186/s13054-015-0763-6 (PMC4355459; doi:10.1186/s13054-015-0763-6)
Supplement: Additional file 3: — Example of Type II double triggering under NAVA. [file 13054_2015_763_MOESM3_ESM.doc]

**Additional File 3: Example of Type II double triggering under NAVA.**

**
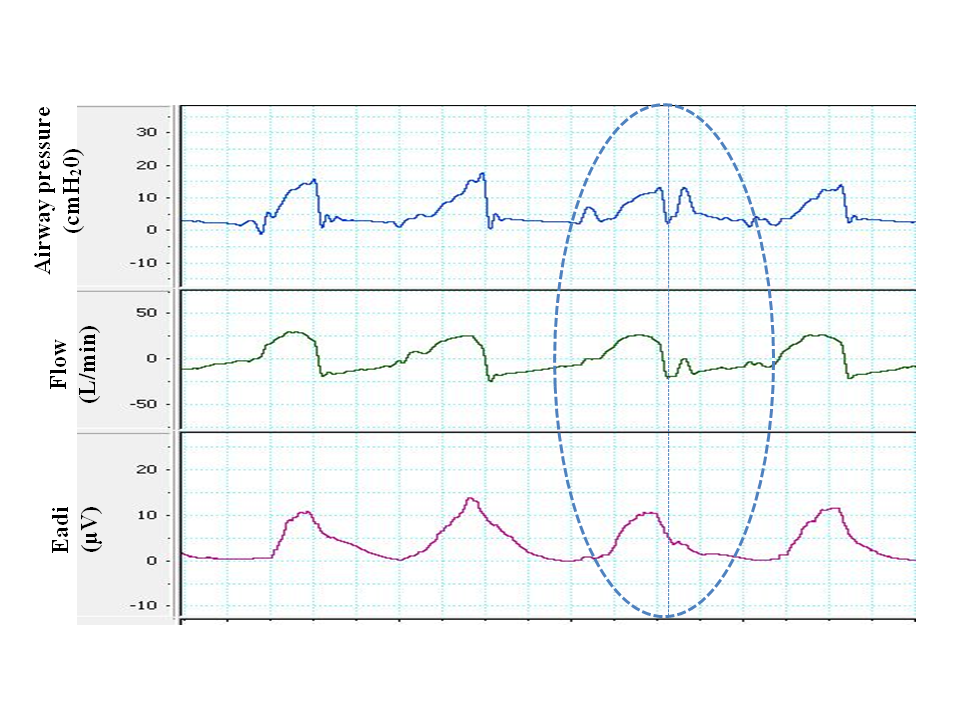
**

*EAdi,* electrical activity of the diaphragm.

Note that the ventilator cycled off when the EAdi dropped to 70% of its peak and re-triggered when cycled off to PEEP.
