# Supplementary material for: Neurally adjusted ventilatory assist and proportional assist ventilation both improve patient-ventilator interaction
Source: Crit Care. 2015 Feb 25;19(1):56. doi: 10.1186/s13054-015-0763-6 (PMC4355459; doi:10.1186/s13054-015-0763-6)
Supplement: Additional file 4: — Inspiratory pressure (cmH 2 O) over PEEP for each patient under the three modes and three assist levels. [file 13054_2015_763_MOESM4_ESM.doc]

**Additional File 4. Inspiratory pressure (cmH_2_O) over PEEP for each patient under the three modes and three assist levels**

| **Patient**  **No.** | **PSV_50_** | **NAVA_50_** | **PAV_50_** | **PSV_100_** | **NAVA_100_** | **PAV_100_** | **PSV_150_** | **NAVA_150_** | **PAV_150_** |
| --- | --- | --- | --- | --- | --- | --- | --- | --- | --- |
| 1 | 13 | 19 | 12 | 21 | 24 | 15 | 29 | 26 | 20 |
| 2 | 9 | 9 | 7 | 13 | 15 | 11 | 16 | 18 | 16 |
| 3 | 9 | 13 | 9 | 17 | 20 | 15 | 25 | 22 | 25 |
| 4 | 7 | 11 | 13 | 12 | 15 | 21 | 18 | 17 | 31 |
| 5 | 8 | 17 | 14 | 16 | 23 | 16 | 24 | 34 | 19 |
| 6 | 8 | 12 | 13 | 16 | 17 | 21 | 23 | 20 | 28 |
| 7 | 9 | 12 | 8 | 16 | 9 | 14 | 23 | 19 | 20 |
| 8 | 9 | 15 | 15 | 17 | 25 | 17 | 24 | 35 | 18 |
| 9 | 10 | 17 | 12 | 18 | 30 | 17 | 25 | 34 | 20 |
| 10 | 10 | 15 | 12 | 18 | 18 | 17 | 25 | 23 | 27 |
| 11 | 8 | 13 | 12 | 16 | 16 | 16 | 24 | 19 | 20 |
| 12 | 9 | 21 | 11 | 18 | 28 | 14 | 26 | 28 | 27 |
| 13 | 7 | 17 | 13 | 9 | 22 | 17 | 14 | 32 | 28 |
| 14 | 20 | 27 | 27 | 30 | 35 | 36 | 35 | 34 | 38 |
| 15 | 7 | 21 | 12 | 13 | 36 | 31 | 19 | 32 | 23 |
| 16 | 7 | 8 | 7 | 8 | 14 | 8 | 12 | 20 | 8 |

*PSV,* pressure support ventilation*; NAVA,* neurally adjusted ventilatory assist; *PAV*, proportional assist ventilation.

Level_100_ is a medium assistance level set to obtain a VT of 6–8 ml.kg^-1^ ideal body weight. Level_50_ is a low assistance level defined as level_100_ decreased by 50%. Level_150_ is a high assistance level defined as level_100_ increased by 50%.
