# Supplementary material for: Neurally adjusted ventilatory assist and proportional assist ventilation both improve patient-ventilator interaction
Source: Crit Care. 2015 Feb 25;19(1):56. doi: 10.1186/s13054-015-0763-6 (PMC4355459; doi:10.1186/s13054-015-0763-6)
Supplement: Additional file 5: — Impact of ventilator mode and assistance level on the coefficients of variation of neural respiratory rate, tidal volume, and peak electrical activity of the diaphragm (EAdi max ). [file 13054_2015_763_MOESM5_ESM.doc]

**Additional File 5. Impact of ventilator mode and assistance level on the coefficients of variation of neural respiratory rate, tidal volume and peak electrical activity of the diaphragm (EAdi_max_**)

|  | **PSV** | **NAVA** | **PAV** |
| --- | --- | --- | --- |
| Coefficient of variation of Tidal Volume |  |  |  |
| level_50_ | 12.1 (11.0-19.1) | 20.7 (16.4-27.3) * | 17.7 (13.8-29.0) |
| level_100_ | 12.6 (10.7-18.5) | 19.1 (14.0-31.2) * | 21.4 (16.0-28.7) * |
| level_150_ | 12.7 (10.7-19.6) | 23.1 (19.2-29.4) * | 21.9 (14.5-34.2) * |
| Coefficient of variation of inspiratory time |  |  |  |
| level_50_ | 11.4 (7.3-18.1) | 12.7 (6.2-27.4) | 16.8 (7.2-28.2) |
| level_100_ | 9.7 (7.7-13.6) | 20.4 (10.6-26.7) * | 18.3 (7.2-34.5) * |
| level_150_ | 13.9 (8.9-31.7) | 19.4 (10.6-29.3) | 14.0 (5.5-25.0) |
| Coefficient of variation of neural respiratory rate |  |  |  |
| level_50_ | 10.3 (9.3-20.1) | 11.3 (7.8-22.4) | 15.1 (8.2-29.7) |
| level_100_ | 12.4 (8.7-15.8) | 18.0 (11.2-25.9) | 17.3 (6.4-29.9) |
| level_150_ | 12.9 (9.5-30.6) | 18.4 (12.6-34.3) | 13.7 (6.6-21.0) |
| Coefficient of variation of EAdi_max_ |  |  |  |
| level50 | 26.5 (24.2-36.7) | 29.0 (24.0-38.7) | 28.0 (19.5-32.8) |
| level100 | 34.0 (23.2-51.0) | 28.0 (21.0-38.2) | 26.0 (17.7-35.3) |
| level150 | 38.0 (27.5-62.0) | 31.0 (21.5-39.7) | 29.0 (23.0-41.2) * |

*PSV,* pressure support ventilation*; NAVA,* neurally adjusted ventilatory assist; *PAV*, proportional assist ventilation.

Level_100_ is a medium assistance level set to obtain a VT of 6–8 ml.kg^-1^ ideal body weight. Level_50_ is a low assistance level defined as level_100_ decreased by 50%. Level_150_ is a high assistance level defined as level_100_ increased by 50%.

* p <0.05 with PSV; data are expressed as median (interquartile range).
