# Supplementary material for: Neurally adjusted ventilatory assist and proportional assist ventilation both improve patient-ventilator interaction
Source: Crit Care. 2015 Feb 25;19(1):56. doi: 10.1186/s13054-015-0763-6 (PMC4355459; doi:10.1186/s13054-015-0763-6)

**Additional File 6: Distribution of the inspiratory trigger delays per mode**

*PSV,* pressure support ventilation*; NAVA,* neurally adjusted ventilatory assist; *PAV*, proportional assist ventilation.


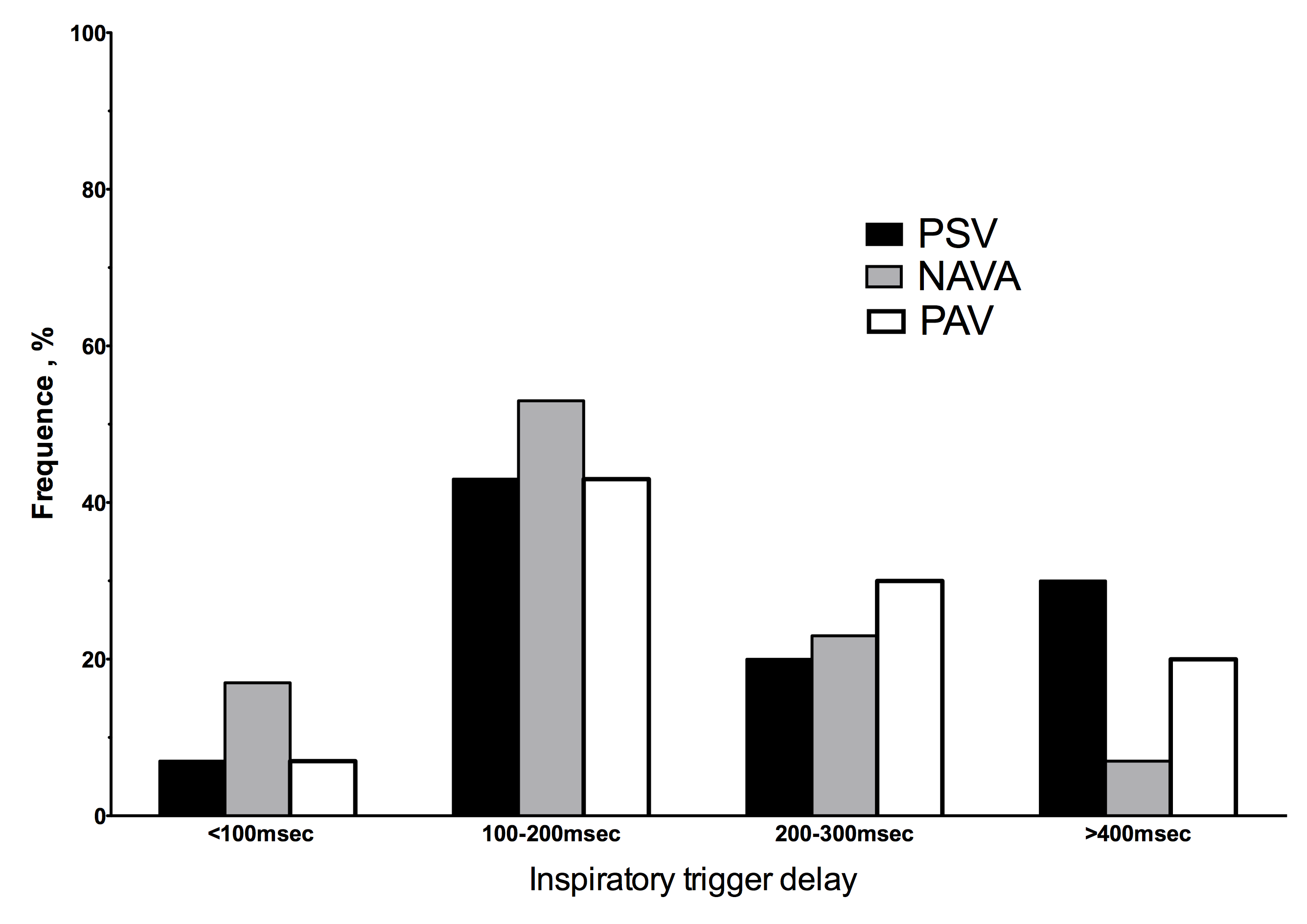

Supplement: Additional file 6: — Distribution of the inspiratory trigger delays per mode. [file 13054_2015_763_MOESM6_ESM.doc]
