# Supplementary material for: Neurally adjusted ventilatory assist and proportional assist ventilation both improve patient-ventilator interaction
Source: Crit Care. 2015 Feb 25;19(1):56. doi: 10.1186/s13054-015-0763-6 (PMC4355459; doi:10.1186/s13054-015-0763-6)
Supplement: Additional file 7: — Impact of ventilator mode and level of assistance on gas exchange. [file 13054_2015_763_MOESM7_ESM.doc]

**Additional File 7. Impact of ventilator mode and level of assistance on gas exchange.**

|  | **PSV** | **NAVA** | **PAV** |
| --- | --- | --- | --- |
| pH |  |  |  |
| level_50_ | 7.38 (7.36-7.43) | 7.39 (7.35-7.44) | 7.38 (7.33-7.44) |
| level_100_ | 7.39 (7.37-7.46) | 7.43 (7.35-7.46) | 7.38 (7.35-7.44) ^£^ |
| level_150_ | 7.41 (7.39-7.47) | 7.43 (7.36-7.47) | 7.39 (7.35-7.48) |
| PaO_2_ |  |  |  |
| level_50_ | 78 (74-91) | 79 (73-101) | 80 (75-90) |
| level_100_ | 81 (74-96) | 80 (71-95) | 78 (74-88) |
| level_150_ | 78 (72-94) | 79 (71-92) | 78 (69-92) |
| PaCO_2_ |  |  |  |
| level_50_ | 38 (35-49) | 39 (34-49) | 39 (34-53) |
| level_100_ | 37 (33-45) | 36 (32-47) | 39 (32-48) ^£^ |
| level_150_ | 36 (32-44) | 35 (32-46) | 38 (31-47) |

*PSV,* pressure support ventilation*; NAVA,* neurally adjusted ventilatory assist; *PAV*, proportional assist ventilation.

Level_100_ is a medium assistance level set to obtain a VT of 6–8 ml.kg^-1^ ideal body weight. Level_50_ is a low assistance level defined as level_100_ decreased by 50%. Level_150_ is a high assistance level defined as level_100_ increased by 50%.

Blood gases were obtained in 13/16 patients.

* p <0.05 with PSV; £ p <0.05 with NAVA; data are expressed as median (interquartile

range).
